# Multidiscipline Immunotherapy-Based Rational Combinations for Robust and Durable Efficacy in Brain Metastases from Renal Cell Carcinoma

**DOI:** 10.3390/ijms22126290

**Published:** 2021-06-11

**Authors:** Hye-Won Lee

**Affiliations:** Center for Urologic Cancer, National Cancer Center, Department of Urology, Goyang 10408, Korea; nsproper@naver.com; Tel.: +82-31-920-1719

**Keywords:** brain metastases, renal cell carcinoma, immune checkpoint inhibitors, treatment resistance, tumor immune microenvironment, combination, tyrosine kinase inhibitor, radiotherapy

## Abstract

Advanced imaging techniques for diagnosis have increased awareness on the benefits of brain screening, facilitated effective control of extracranial disease, and prolonged life expectancy of metastatic renal cell carcinoma (mRCC) patients. Brain metastasis (BM) in patients with mRCC (RCC-BM) is associated with grave prognoses, a high degree of morbidity, dedicated assessment, and unresponsiveness to conventional systemic therapeutics. The therapeutic landscape of RCC-BM is rapidly changing; however, survival outcomes remain poor despite standard surgery and radiation, highlighting the unmet medical needs and the requisite for advancement in systemic therapies. Immune checkpoint inhibitors (ICIs) are one of the most promising strategies to treat RCC-BM. Understanding the role of brain-specific tumor immune microenvironment (TIME) is important for developing rationale-driven ICI-based combination strategies that circumvent tumor intrinsic and extrinsic factors and complex positive feedback loops associated with resistance to ICIs in RCC-BM via combination with ICIs involving other immunological pathways, anti-antiangiogenic multiple tyrosine kinase inhibitors, and radiotherapy; therefore, novel combination approaches are being developed for synergistic potential against RCC-BM; however, further prospective investigations with longer follow-up periods are required to improve the efficacy and safety of combination treatments and to elucidate dynamic predictive biomarkers depending on the interactions in the brain TIME.

## 1. Introduction

Renal cell carcinomas (RCC) comprise a heterogeneous histologic subtype of malignant neoplasms arising from the nephron, and widely vary with respect to the underlying pathogenesis, genomic/molecular characteristics, and clinical treatment, including the susceptibility to conventional therapeutics [1,2,3,4,5,6,7,8]. Sequential events in the molecular etiopathogenesis of Von Hippel–Lindau (VHL)-inactivated metastatic RCC (mRCC) can be summarized as constitutive activation of the hypoxia-inducible factor (HIF) pathway due to loss of VHL activity, fostering global changes in the metabolome, genome, and epigenome in apoptotic, cell cycle regulatory, and mismatch repair pathways, angiogenesis, metabolic adaptations, and immune evasion [1,2,3,4,5,9,10].

Systemic therapy against mRCC has evolved significantly over the past two decades [11,12,13,14]. Vascular endothelial growth factor (VEGF)-tyrosine kinase inhibitors (TKIs) (e.g., sunitinib, pazopanib, axitinib, tivozanib, lenvatinib, and cabozantinib) or mammalian target of rapamycin (mTOR) pathway inhibitors (e.g., everolimus and temsirolimus) were used in the 2000s for mRCC, either combined or as a monotherapy [11,12,13,14]. A validation study of the International Metastatic RCC Database Consortium (IMDC) system revealed that the median overall survival (OS) significantly improved in the 2000s as compared to that observed in the cytokine era (favorable risk: 43.2 months, intermediate risk: 22.5 months, and poor risk: 7.8 months) [15]. Unfortunately, the 5-year OS rate for mRCC remains under 30% despite the TKI-based approach [11,12,13,14].

## 2. Clinical Implications and Unmet Needs of Brain Metastases from RCC

Brain metastases (BM) is generally associated with a very poor prognosis and high degree of morbidity, requiring urgent multidisciplinary care, and is relatively unresponsive to conventional systemic therapy [11,16,17,18,19,20,21,22,23,24,25]. BM is also a serious condition that causes headaches, focal neurological deficits, altered mental status or gradual cognitive impairment, epileptic seizures driven by increased intracranial pressure by vasogenic edema, or alterations in cerebrospinal fluid (CSF) flow, thereby impairing the quality of life (QOL) [11,17,18,19,25,26,27]. Unfortunately, BM is not a rare finding in mRCC (8%–15%), and its prevalence has increased in the past two decades [16,17,18,19,20,21,22]. The median OS of RCC patients with BM (RCC-BM) is only 5–8 months [16,17,18,19,20,21,22]. Therefore, early detection and effective treatment of BM is an unmet medical need for mRCC [11,17,19,25]. Improved clinical outcomes of extracranial metastases by the introduction of TKIs and immune checkpoint inhibitors (ICIs) has led to the adoption of improved imaging techniques for BM, thereby increasing awareness of the benefit of brain screening [11,17,18,19,20,27].

As advances in both local and systemic therapies provide better survival outcomes, mRCC patients with solitary BM and good performance status (PS) can benefit from early detection of asymptomatic BM [11,19,25,28]. Nevertheless, current surveillance guidelines for patients with RCC do not recommend brain imaging, unless BM is clinically suspected [11,29,30]. A large mRCC cohort study to investigate the rate of incidental BM highlighted that a relevant proportion of patients with mRCC (4.3%) may harbor occult BM through brain imaging as a part of eligibility assessments for clinical trial [16]. These data suggested that the risk of asymptomatic brain involvement extends to those with favorable risk features per IMDC risk assessment [16,19]. Sarcomatoid dedifferentiation, T2-4 disease, tumor size >10 cm, regional node involvement, and thoracic and osseous sites of extracranial disease, initial presence with metastatic disease at diagnosis and disease progression during first-line therapies were independent BM-associated risk factors for mRCC [16,19,20,25,31,32]. Consistent evaluation of risk and identification of highly sensitive and accurate algorithmic screening approaches are required to characterize mRCC patients with a considerably high propensity for BM, given that early detection may improve clinical outcomes and decrease the potential risks of aggressive multimodality treatment in patients with mRCC [11,16,19,25,28,31].

Unfortunately, brain lesions may be detected only after establishment of a microenvironment supportive of tumor growth and visible proliferation using magnetic resonance imaging (MRI) or computed tomography scans; however, these technologies are not sensitive enough to detect very early metastases [11,19,25,28]. Alternative approaches that can enable early diagnoses of BM are thus being explored based on liquid biopsy of circulating tumor DNA obtained from the CSF [33]. The implications of liquid biopsies for BM are notable, as they facilitate early detection and molecular profiling of a brain lesion to initiate the most appropriate treatment. Finally, prognostic factors are important for determining the optimal treatment modality for RCC-BM [11].

## 3. Immunosuppressive Pro-Metastatic Brain-Specific Tumor Microenvironment (TME)

The immune landscape of RCC-BM is less characterized than those of primary brain cancers [34,35]. The central nervous system (CNS) is protected by several functional barriers, including the blood–brain barrier (BBB) and blood–CSF barrier [34,35,36,37]. The BBB consists of endothelial cells with low transcytosis rates and high expression of efflux pumps that are connected by continuous tight junctions [34,35,36,37]. In addition, two basement membranes, embedded pericytes, and astrocytic terminal processes contribute to the BBB functions [34,35,36,37]. By comparison, the blood–CSF barrier is formed by choroid plexus epithelial cells that are connected via tight junctions, with choroid plexus capillaries having fenestrations and intercellular gaps that enable the free movement of molecules between these compartments [34,35,36,37]. Diffusion restriction of systemic agents into the CNS is considered a potential obstacle for intracranial efficacy of multiple TKIs and ICIs [16]. However, in patients with BM, the BBB is leaky and is substituted by a blood–tumor barrier (BTB) with a wider fenestration, leading to a higher efflux of fluid [17,34,37,38,39].

The development of BM disrupts the BBB damaged by a prominent neuroinflammatory response and anti-tumor treatment, such as surgery and/or radiotherapy, and is often characterized by abnormal vascular sprouting, allowing an influx of circulating myeloid and lymphoid cells, which are generally absent in the brain parenchyma, into the CNS [17,27,35,37,38]. The composition of the brain-specific immunosuppressive TME revealed cancer-specific enrichment of immune cells with pronounced differences in proportional abundance of microglia, infiltrating monocyte-derived macrophages, neutrophils, and T cells, playing a major role in BM progression, and creating a multitude of potential targets [38].

CNS myeloids, microglia and border-associated myeloid cells (BAMs), vitally contribute to brain homeostasis and diseases [38,40,41]. At homeostasis, microglia are the brain’s equivalent of tissue-resident macrophages, representing 5%–15% of adult brain cells [37,40,41]. BAMs reside specifically in the meninges, choroid plexus, or perivascular macrophages associated with blood vessels [34,37,38,42]. Microglia and BAMs have different gene expression signatures, with BAMs being characterized by high CD38 and major histocompatibility complex (MHC) class Ⅱ, thus supporting their role as antigen presenting cells [34,38,42]. In addition, peripheral bone marrow-derived myeloid cells (BMDMs) may infiltrate the brain parenchyma and contribute to neuroinflammation [38,42]. CNS myeloids promote BM via chemokine (C-X-C motif) ligand 10 (Cxcl10) signaling and negative immune checkpoints that foster an immune suppressive niche, indicating blocking V-type immunoglobulin domain-containing suppressor of T cell activation (VISTA) and programmed cell death ligand 1 (PD-L1) signaling as an effective immune strategy [34,35,37,38,42].

Another predominant (up to 30% of the tumor mass) immune subset of BM TME are tumor-associated macrophage (TAM)-peripheral bone marrow-derived myeloid cells (BMDMs) [34,35,37,38,42]. They can be localized in the advancing tip of the tumor, blood vessels, or perinectrotic areas, where they play a role in tumor cell motility, establishment of metastatic niche, or angiogenesis, respectively [34,35,37,38,42]. Tissue-invading TAM-BMDMs with complex multifaceted phenotypes show a distinctive signature trajectory, revealing tumor-driven instructions along with contrasting tumor-infiltrating lymphocyte (TIL) activation and exhaustion [34,38,42]. When stimulated and reprogrammed by cancer cells, TAM-BMDMs can secrete immunosuppressive biomolecules, including transforming growth factor-β (TGF-β), interleukin (IL)-10, and arginase [37,43]. As TAM-BMDMs are implicated in BM promotion and exhibit gene signatures that are associated with wound healing, antigen presentation, and immune suppression [34,35,38,42], selective depletion or blockade of TAM-BMDM recruitment could lead to effective T cell activation and execution of anti-tumor effector functions [27,34,38,42].

A range of lymphoid cells, including B and T cells, as well as innate lymphoid, natural killer (NK), and NK T cells, may be found within the CSF of the meninges, choroid plexus, and ventricles, although they are absent from the brain parenchyma [27,37,40]. RCC-BM leads to a moderate T cell influx, and T cells are predominantly localized within the stromal compartments of the tumor [35]. Tumor cells may also produce indolamine 2,3-dioxygenase (IDO), which stimulates the accumulation of regulatory T cells (Treg) and suppresses T cell activity by depleting tryptophan from the TME [37,38,42,44]. Importantly, the presence of PD-L1^+^ TAMs has been correlated with Treg frequencies in several solid tissue tumors [38,42,45]. Tregs secrete IL-10, IL-4, and IL-13, which may trigger the development of TAMs with immunosuppressive properties and suppression of cytotoxic CD8^+^ T cell responses [38,42,46]. The major changes in T cell activation in BM are the activation and exhaustion of CD8 tissue resident memory and effector memory subsets, displaying high amounts of both co-stimulatory and co-inhibitory molecules, as well as proliferation markers [35,38,42].

## 4. Multimodal ICI-Based Therapeutic Strategies for RCC-BM

RCC-BM poses unique clinical challenges because treatment of BM is complex, and a variety of factors, including anticipated patient survival, competing risks, and long-term toxicities should be considered while selecting the appropriate treatment strategy [11,17,47]. The brain, being a vital organ, is unable to regenerate upon damage, thus accounting for major limitations for therapy [11,17,47,48]. For instance, neurosurgery cannot always be performed, and radiotherapy has the risk of irreversibly limiting brain plasticity, which could evolve into a potentially lethal radionecrosis [48]. Three key indicators favor a good prognosis and thus more aggressive treatment: a KPS score > 70, age < 65 years, and controlled extracranial metastases [47,49].

The current approach for RCC-BM typically includes surgery (pathologic diagnosis and cerebral decompression) versus standalone radiotherapy and/or systemic therapies, with the overall goal of selecting the optimal treatment for an individual patient to maximize QOL and survival outcomes [17,47,48,49,50]. Surgery and radiation are the mainstays of therapy and have proven neurological and palliative benefits [17,47,49,50,51]. Medical therapies for RCC-BM can be divided into two broad categories: symptomatic management and tumor-targeting therapies. Corticosteroids, such as dexamethasone, represent the main symptomatic treatment in addition to pain medications because of their minimal mineralocorticoid effect and control intracerebral edema in BM [17]; however, the beneficial effects of steroids are not permanent, and a rapid taper is typically recommended to minimize drug-related adverse effects [17]. In addition, increased understanding of the role of immunosuppression in the pathophysiology of metastatic diseases reveals the potential harm of steroid-associated immunosuppression, thereby encouraging minimal steroid exposure and alternatives to steroid therapy [17].

### 4.1. ICIs Based on T Cell Exhaustion in RCC-BM 

Although most patients with RCC-BM are excluded from important clinical trials because of poor prognosis and few validated treatment guidelines [11,48], this trend is diminishing given the increasing importance of clinical significance and a better knowledge of the underlying pathogenesis. The remaining majority of systemic therapies for RCC-BM dramatically changed with the introduction of ICIs and TKIs based on complex microenvironmental niche–tumor interactions, neuroinflammatory cascades, and neovascularization involved in establishing a new BM [11,17,47,48]. The richness and activation of BM TMEs regarding cellular subtypes, frequencies, and functional states parallels their favorable clinical response to ICIs [42]. Checkpoint interactions, such as PD-1:PD-L1, CTLA4:B7-1/2, T-cell immunoglobulin and mucin domain-3 (TIM-3):Galectin-9, and lymphocyte activation gene-3 (LAG-3):MHC class Ⅱ, play an important role in immune evasion of cancers [1]. Drug Administration (FDA) for mRCC include those that block co-inhibitory molecules, such as cytotoxic T-lymphocyte activating protein-4 (CTLA-4), PD-1, and PD-L1, thus facilitating T cell effector function and anti-tumor response [52,53].

Costimulation with CD28 or 4-1BB can increase anti-tumor activity [54,55]. CD28 costimulation can increase T cell anabolic metabolism, while the CD28 family members PD-1 and CTLA4 suppress T cell metabolic reprogramming [54]. CTLA4 inhibits CD28 signaling and PI3K/Akt/mTORC1 signaling, resulting in decreased glycolysis and mitochondrial oxidative capacity [56]. Blocking the negative regulators of PD-1 and CTLA4 that impair CD28 signaling to inhibit T cell release facilitates anti-tumor activity [54]. CD8^+^ T cells continuously formulate their exhaustion states on account of exposure to suppressive gradients in the TME [57]. T cell exhaustion is the conversion of the state of CD8^+^ T cells from antineoplastic to immune-functionally impaired due to long-term persistence of tumor antigens and/or the suppressive TME [58,59]. The exhausted CD8^+^ T-cell phenotype has been associated with an increased risk of tumor progression [60,61], increased dysfunctional dendritic cells (DCs) [62], and elevated numbers of immune cells, namely M2-polarized macrophages, resting mast cells, resting memory CD4^+^ T cells, and CD4^+^ Foxp3^+^ Tregs [60,63,64,65]. Coinhibitory receptors, such as PD-1 and CTLA-4, are traditionally envisioned as exhaustion markers of T cells [59,66], which is the theoretical ICB.

Additionally, the prognostic impact of exhausted CD8^+^ T cell infiltration in mRCC is only through stratification into specific subgroups [60,62,64,67,68,69,70]. For example, CXCL13^+^ CD8^+^ T cells exhibit elevated levels of markers, such as PD-1, Tim-3, T cell immunoreceptor with Ig and ITIM domains (TIGIT) and CTLA-4, higher Ki-67 expression, and lower levels of activated markers, such as tumor necrosis factor α (TNF-α) and interferon γ (IFN-γ) [67,71]. Furthermore, the abundance of intratumoral CXCL13^+^ CD8^+^ T cells was positively correlated with immunoevasive TME accompanied by increased T helper 2 cells, TAMs, CD4^+^ Foxp3^+^ Tregs, and decreased NK cells [67]. The HIF-1-TGF-β pathway might serve as a crucial molecule in connecting CXCL13^+^ CD8^+^ T cells and TME, [67,72,73,74]. Neoantigen reactivity is coupled to a CXCL13-secreting ‘‘exhausted’’ phenotype, possibly induced by chronic TCR signaling [75]. The selective expression of CCR5 and CXCL13 in neoantigen-specific T cells further suggests that a key feature of ICI-responsiveness is the ability to sustain ongoing priming and recruitment of tumor reactive T cells supported by CXCR5^+^ lymphocytes [76,77]. Interestingly, patients with higher numbers of CD39^+^ CD8^+^ T cells showed improved responses to sunitinib, a multi-TKI, suggesting that evaluation of the exhausted phenotype for CD8^+^ T cells may help in clinical decision making or therapy selection [61].

Many receptors in the immunoglobulin superfamily (such as CD28, and inducible T cell co-stimulator) and TNF receptor superfamily (TNFSF) exert costimulatory actions [78]. TNFSRF9 is thought to be an antigen stimulation-inducible co-stimulatory receptor, which is transiently expressed on activated CD8^+^ T, activated CD4^+^ T, and NK cells [64,79,80]. Co-stimulatory signaling mediated by TNFRSF9 promotes T cell proliferation, secretion of cytokines, resistance to activation-induced cell death, and development of memory T cells [80]. TNFRSF9^+^ CD8^+^ T cells possess both exhaustion (PD-1, TIM-3, CTLA-4, and TIGIT) and effector phenotype (IFN-γ, granzyme B, and Ki-67) [79]. This dual phenotype of TNFRSF9^+^ CD8^+^ T cells indicates that these cells may not be terminally exhausted; however, they could respond to ICB [79]. The functional status of TNFRSF9^+^ CD8^+^ T cells might partly result from the complicated interactions among immune cells (helper T cells, CD8^+^ T cells, and myeloid cells) within the tumor, and high enrichment of TNFRSF9^+^ CD8^+^ T cells could be a predictor of immunotherapy and a novel therapeutic target in mRCC [79].

### 4.2. ICIs Based on Targeting Immunometabolomics in RCC-BM

RCC is essentially a metabolic disease characterized by a reprogramming of energetic metabolism, and many genes that are mutated in RCC encode proteins that have roles in cellular processes regulating oxygen and glucose consumption [54]. In particular the metabolic flux through glycolysis is partitioned [81,82,83,84], and mitochondrial bioenergetics and oxidative phosphorylation are impaired, as well as lipid metabolism [82,85,86]. In addition, RCC is one of the most immune-infiltrated tumors [87,88]. Emerging evidence suggests that the activation of specific metabolic pathway have a role in regulating angiogenesis and inflammatory signatures [89,90]. Features of the TME heavily affect disease biology and may affect responses to systemic therapy [91]. VHL mutations that occur in mRCC increased transcriptional activity of its target genes, such as *VEGF*, glucose transporter 1, and erythropoietin, independent of oxygen levels, promoting angiogenesis, and immunosuppression [54]. The complexity of cellular interactions and depletion of available nutrients may create an environment of nutrient competition for T cells, and buildup of waste products that may impair T cells [92]. RCC-BM demonstrated metabolic changes leading to alterations in pathways associated with energy metabolism and oxidative stress, as well as the accumulation of immunosuppressive metabolites, such as tryptophan (TRP) [54,92]. Enhanced activity across an array of interconnected oncogenic signaling networks centered on the PI3K-AKT pathway represents a generalizable feature across different BM histologies [92].

The analysis of metabolic pathways intrinsic to immune cell types, also known as immunometabolism, could identify markers of immune function based on the distinct metabolic requirements of these cells at each stage of differentiation [93]. At the single-cell level, costimulation shifted the percentage of cells from a baseline resting state into two primary branches: one that was enriched in IL-2 signaling and glycolysis and another that exhibited pathways of glycolysis, oxidative phosphorylation, and Myc signaling [54]. This bioenergetic switch is consistent with the known Myc regulation of metabolic reprogramming during T cell activation [54]. Activation, together with signaling through the costimulatory molecule CD28, augments signaling through the PI3K/Akt/mTORC1 pathway to increase glucose and mitochondrial metabolism, and enable robust proliferation and effector function [94,95].

Metabolic reprogramming dictates the fate and function of stimulated T cells and microenvironment of tumors coupled with chronic exposure to neoantigens can impair the metabolism of TILs [54,96,97]. Stimulated T cells are highly dependent on metabolic reprogramming from catabolic oxidative metabolism to anabolic metabolism with elevated glucose consumption and aerobic glycolysis to develop effector functions [98,99,100]. T cell activation leads to increased Myc and PI3K/Akt/mTORC1 signaling activity to promote glucose uptake and mitochondrial metabolism for growth and energetics, and to regulate signaling and gene expression pathways [95,101,102]. CD8^+^ T cells in RCC can be subject to metabolic barriers that lead to adaptations, such as reduced ability to absorb glucose for downstream glycolysis, fragmented and functionally altered mitochondria with low respiratory capacity, and elevated production of reactive oxygen species (ROS) [97,103]. These changes are critical for effector T cell function, as CD8^+^ PD-1^+^ cells subject to inhibition of glucose metabolism fail to develop into effector subsets and have a reduced capacity to favor suppressive Treg fates [54,103]. RCC CD8^+^ TILs have altered metabolic and functional parameters, suggesting reduced metabolism and failure of antigen receptor stimulation to activate a predominant effector memory phenotype [54]. 

In addition, PD-1 signaling suppresses T cell metabolic reprogramming by inhibiting glycolysis and promoting lipolysis and fatty acid oxidation (FAO) [54,56]. While CD8^+^ RCC TIL gene expression exhibits classical markers of chronic stimulation and enrichment of metabolic pathways, including FAO, glycolysis, and cholesterol homeostasis, a large portion of cells could be stimulated to reprogram metabolism and induce effector functions [54]. The link between very long chain fatty acid-containing lipids and response to ICI in RCC can be explained by enhanced peroxisome signaling in activated T cells, which leads to a metabolic switch to fatty acid catabolism [104]. Lastly, increased conversion of TRP to kynurenine by IDO leads to inhibition of T cell function and is involved in the regulation of the immunosuppressive TME of mRCC [54,105].

## 5. ICI Monotherapy for mRCC

Targeting immune suppression using ICIs has resulted in clinical responses in some patients with mRCC, and combinatorial approaches involving checkpoint blockade are now the standard of care in patients with advanced mRCC [106]. Elucidation of the mechanisms that underlie responses or resistance to ICIs will enable the rational development of combinatorial strategies aimed at improving the efficacy of these therapies [106]. 

### 5.1. Nivolumab

Nivolumab, a human IgG4 monoclonal antibody (mAb) that blocks PD-1, has been approved as a second-line treatment after disease progression during TKI therapy [1,107,108]. Nivolumab demonstrated activity in patients with mRCC when used as a monotherapy in both phase I and phase II clinical trials [107,109,110,111]. Objective responses in these trials ranged from 20%–27%, with certain durable responses. In the first phase Ib biomarker study (CheckMate 003; NCT00730639) of nivolumab monotherapy (1, 3, or 10 mg/kg every two weeks), the objective response rate (ORR) was 27% (9/33), and toxicities with grade ≥ 3 were observed in 14% patients with mRCC who had received prior systemic therapy (non-ICIs) [109]. Subsequently, nivolumab was evaluated in a randomized dose-ranging phase II trial (CheckMate 010; NCT 01354431), recruiting 168 patients with mRCC who had received prior VEGF therapy [107]. ORRs ranged from 20%–22% for the administered doses of 0.3, 2, and 10 mg/kg without any significant dose–response effects. A similar response was observed for progression-free survival (PFS) and adverse events (AEs) [107].

These encouraging results were confirmed in a phase Ⅲ open-label trial (CheckMate 025; NCT01668784) comparing nivolumab and the mTOR inhibitor everolimus in 821 patients with mRCC administered prior treatment with VEGF [108]. Nivolumab therapy was associated with superiority and elicited a significantly improved OS than everolimus. This survival advantage was notably associated with fewer severe (grade ≥ 3) AEs in patients treated with nivolumab (19%) than everolimus (37%), and higher QOL scores on the Functional Assessment of Cancer Therapy Kidney Symptom Index-Disease-Related Symptoms scoring algorithm. Recently, updated results from this trial population with a minimum of five years of follow-up have established long-term favorable safety and efficacy for nivolumab monotherapy [108].

Another phase Ⅱ trial (GETUG-AFU 26 NIVOREN) evaluated the efficacy and safety of nivolumab in patients with RCC-BM after TKI therapy [112]. The trial constituted of two cohorts: cohort A comprised patients with previously untreated BM (*n* = 39), and cohort B comprised patients with previous therapy for BM (*n* = 34) [112]. The intracranial response rate was 12% in cohort A [112]; no objective response was reported in patients with brain lesions that were multiple or larger than 1 cm [112]. Patients who received prior focal therapy had a significantly decreased risk of intracranial progression compared to that in patients with untreated BM, suggesting that administration of focal therapy before ICIs should be considered in a specific subgroup of RCC-BM [112]. In conclusion, single-agent nivolumab has limited activity in patients with untreated RCC-BM, who experienced progression after VEGF-targeted therapy. These data highlight the need to pursue dedicated clinical trials in this population and advocate the evaluation of combination strategies using systemic and focal brain therapies.

Pembrolizumab is a highly selective IgG4-kappa humanized mAb that targets PD-1 [47]. The preliminary results of a phase Ⅱ trial (Keynote 247) in patients with advanced or mRCC showed that pembrolizumab monotherapy resulted in an overall ORR of 36% [113]. The safety and efficacy of pembrolizumab monotherapy in treatment-naïve patients with mRCC are also currently being evaluated in a phase Ⅱ clinical trial (KEYNOTE-427; NCT02853344). Preliminary analysis of the cohort enrolling 110 patients with mRCC revealed an ORR of 36.4% with a median follow-up of 12 months at the time of data cutoff [114].

### 5.2. Avelumab, Atezolizumab, and Durvalumab

In addition to targeting the PD-1 receptor on T cells, antibodies targeting PD-L1 (avelumab and atezolizumab) have been developed and are now in clinical trials [115,116,117,118]. Avelumab, a fully human mAb that targets PD-L1, was found to have a favorable safety profile but modest efficacy in a phase Ⅰa trial (NCT10375842) [107] and a randomized phase Ⅱ trial (IMmotion150; NCT01984242) [115,117]. In the IMmotion 150 trial in treatment-naive patients with mRCC, atezolizumab monotherapy resulted in an ORR of 25% [115]. In the JAVELIN solid tumor trial in patients with mRCC, first-line treatment with avelumab monotherapy resulted in an objective response rate of 16% [116].

### 5.3. Ipilimumab

CTLA4, an immune checkpoint protein expressed in activated CD8^+^ T cells and Tregs, has received extensive attention in immunotherapy [118,119]. CTLA4 was markedly correlated with multiple immune checkpoints, which suggested that mRCC patients with high expression of CTLA4 may benefit from ICI combined therapy [120]. Ipilimumab (anti-CTLA-4 mAb) was the first ICI tested in patients with mRCC [121]. A phase Ⅱ trial (NCT00057889) of ipilimumab in patients with mRCC demonstrated low response rates of 12.5% in the high-dose cohort and 5% in the low-dose cohort, with no durable responses [121]. CTLA4 blockade also resulted in a high level of immune-mediated toxicity (grade 3 or 4) in 33% patients [121]. Interestingly, there was a 30% overall response rate among patients experiencing immune-related toxicity and a 0% response rate among those without immune-related toxicity from ipilimumab [121]. Due to the low response rate and high level of toxicity, CTLA4 antibody monotherapy has not been further developed for the treatment of patients with mRCC.

## 6. Combined Regimens Based on ICIs against RCC-BM

As ICIs are superior to conventional TKIs [122], ICI-based therapies are now the standard of care for patients with mRCC [52,53]. A substantial subset of mRCC patients do not respond to ICIs, and patients who initially respond, eventually progress [62,123]. Accordingly, the most potential combination treatment should meet one of the following requirements: (a) ICIs plus immune TME-modifying and anti-angiogenic agents; (b) immune TME-modifying agents plus anti-angiogenic agents; and (c) adoptive T cell therapy plus anti-angiogenic agents [124]. Immunotherapy attenuates immunosuppression and decreases tumor vascularization, and anti-angiogenic drugs reduce the percentage of immunosuppressive cells, thus blocking immune escape. The dominant challenge is to identify an optimal combination of these two treatments and obtain the optimal dosage of combined therapy to magnify the clinical benefits [124]. The therapeutic potential of combinatorial approaches targeting checkpoint molecules alone or in combination with other checkpoint blockers, targeted therapies, or radiation have been extensively explored [16,52,53,125,126,127]. Although trials to date have largely included patients with previously untreated BM, the use of multiple treatments and their efficacy remains largely unexplored.

### 6.1. Combination Strategies: ICI + ICI

CTLA4 is highly related to other immune checkpoints, such as PD-1, PD-L1, LAG3, IDO1, and TIGIT [120,127,128]. The CTLA4 inhibitor combined with other ICIs, namely PD-1 inhibitor nivolumab or LAG3 inhibitor, may yield a better therapeutic response in mRCC [120,129,130]. The non-overlapping effects of anti-CTLA-4 and anti-PD-1 antibodies, inducing predominantly memory T cell proliferation with the former and cytolysis and NK proliferation with the latter, provides the rationale for the combination of these agents even after prior anti-PD-1 failure to induce response [131]. Anti-CTLA4 has been successfully used together with anti-PD-1 as a combinatorial immunotherapy for mRCC, particularly in combination with nivolumab and ipilimumab [112,132,133]. CheckMate 016 (NCT01472081) reported the additive efficacy and maintained the safety profile of combining nivolumab and ipilimumab, followed by nivolumab monotherapy in a phase I dose-escalation study enrolling 194 patients with mRCC [132]. The two dosing regimens included 3 mg/kg nivolumab plus 1 mg/kg ipilimumab (N3I1) and 1 mg/kg nivolumab plus 3 mg/kg ipilimumab (N1I3) [132]. Both dosing arms reported very promising ORRs of 40.4%; however, there was a higher rate of grade ≥ 3 AEs in the N1I3 arm (61.7%) than in the N3I1 arm (38.3%) [132]. 

The phase III CheckMate 214 trial (NCT02231749) demonstrated the efficacy and safety of the nivolumab–ipilimumab combination in treatment-naïve patients with mRCC [133]. Complete response rates (9% vs. 1%), OS (75% vs. 60%), and ORR (42% vs. 27%) were higher after treatment with nivolumab plus ipilimumab than after treatment with sunitinib [133]. Grade ≥ 3 AEs were observed in 46% patients in the ICI arm and 63% patients in the sunitinib arm [133]. Although the survival benefits of nivolumab plus ipilimumab were primarily driven by responses in intermediate/poor-risk patients, long-term disease control was also demonstrated in select favorable risk patients [133]. Based on these findings, the combination of nivolumab plus ipilimumab was approved by the FDA as a standard-of-care frontline treatment of intermediate-risk and poor-risk patients with RCC [133]. Unfortunately, CheckMate 214, which evaluated the combination of nivolumab and ipilimumab as first-line therapy for mRCC, excluded those with BM [133]; however, the ongoing CheckMate 920 phase III b/4 clinical trial included patients with RCC-BM [132]. The ORR was 28.6% (95% CI: 13.2–48.7), and the median PFS was 9.0 months, indicating that the ICI combination treatment may also be promising in RCC-BM patients, with an acceptable safety profile. In summary, dual immune checkpoint inhibition with nivolumab plus ipilimumab, now approved in the front-line treatment for intermediate/poor IMDC risk groups, may benefit patients with BM [47,112,132,133].

Building on the success of antibodies targeting PD-1 and CTLA4 immune checkpoints, multiple innovative immunotherapies are currently in clinical development for the treatment of patients with RCC, including ICIs with novel targets, co-stimulatory pathway agonists, modified cytokines, metabolic pathway modulators, cell therapies, and therapeutic vaccines to overcome resistance [134,135,136,137]. Although the number of trials targeting these immune modulators is increasing, approval for use of these approaches in the clinic has not been granted, either owing to a lack of clinical benefit or because they are still in the early stages of clinical testing. A better understanding of the regulatory mechanisms underlying immune infiltration and activation will likely lead to improved incorporation of immune therapies into the therapeutic landscape for RCC-BM [16,17]. For example, in ICIs, it is not well characterized whether the antibodies penetrate the lesions or reprogram immune cells systemically [17]. Another issue that requires resolution is pseudoprogression, a potentially fatal, intense inflammatory response that can mimic rapid tumor progression [17]. Systemic toxicity with immunotherapies is also a concern, with mild toxicity rates as high as 50% in phase Ⅱ studies, although rates of grade 3 or 4 toxicities were largely <10% [17].

### 6.2. Combination Strategies: ICI + TKI 

Although targeting the VEGF pathway alone resulted in drug resistance and a few durable responses [138], the anti-angiogenic TKIs sunitinib, sorafenib, pazopanib, and cabozantinib have demonstrated some efficacy in patients with BM [11,139,140,141,142,143,144,145]. Gore et al. [146] reported results from an open-label expanded access program on sunitinib for 4564 patients with mRCC from 52 countries. Their report supports the clinical activity of sunitinib in RCC-BM; however, prospective randomized trials led to the conclusion that sunitinib has limited efficacy, although it is acceptably tolerable [139]. The RECORD1 trial and REACT study demonstrated the safety of everolimus in patients previously treated with TKIs [147,148]. These two trials included a subgroup with RCC-BM [147,148]. The safety of temsirolimus for neurologically stable RCC-BM with previous local treatment has also been demonstrated in the ARCC trial [149]. Collectively, the outcomes of first-generation TKIs are not encouraging [11]. However, evidence about their safety would justify their use as first-line treatment in small and asymptomatic BM as part of a multidisciplinary approach and after radiotherapy for stable diseases [11].

Furthermore, angiogenic factors such as VEGF drive immunosuppression in the TME by inducing vascular abnormalities, suppressing antigen presentation and immune effector cells, or augmenting the immunosuppressive activity of Treg, myeloid-derived suppressor cells (MDSCs), and TAMs [47,124,150]. DC immaturity occurs when VEGF binds to VEGF receptor 2 (VEGFR2), thus decreasing antigen presentation and increasing PD-L1 expression in DCs [124]. In addition to inducing angiogenesis by stimulating the generation of blood vessels, VEGF promotes immunosuppression by enhancing the influx of suppressive cell types, such as MDSCs, TAMs, and Tregs into the TME, directly modulating the activity of MDSCs and Treg cells, and inhibiting the maturation of DCs [151]. Subsequently, VEGF downregulates T cell function by blocking CD4^+^ and CD8^+^ cell maturation [124]. In turn, immunosuppressive cells produce VEGF-related proangiogenic cytokines or VEGFR-related expression to weaken the anti-angiogenic agents [124], thereby creating a vicious cycle of suppressed anti-tumor immunity [150]. Based on the cross-talk between immune regulation and angiogenetic modulation, combinations of ICIs and anti-angiogenic TKIs are currently expected to synergistically enhance therapeutic efficacy [124].

Improved anti-tumor immunity in the brain may also be mediated by combinations of antiangiogenic agents and ICIs [150,151]. Recently, the FDA approved two ICIs plus TKI combinations for mRCC: pembrolizumab plus axitinib and avelumab plus axitinib as first-line treatment in untreated intermediate and poor-risk subsets [1,16,53,126,133,150,152]. Both combinations were tested against the TKI sunitinib in large, randomized, multi-center trials and demonstrated improved median PFS with a 4–5-to month margin and superior overall ORR [1,122,126,152]. Promising data from trials confirm a survival benefit with the combination of cabozantinib plus nivolumab and pembrolizumab plus lenvatinib as first-line treatment for mRCC [53,153].

Axitinib plus pembrolizumab and axitinib plus avelumab are considered the standard of care for advanced RCC, irrespective of the IMDC risk status [16,126,133,152]. The first approved regimen includes avelumab in combination with axitinib [126]. Another first-line combination is ICI + TKI; however, pivotal trials, such as KEYNOTE-426 and JAVELIN Renal 101, excluded those with active BM [126,152]. The JAVELIN Renal 101, a randomized phase III trial of avelumab (10 mg/kg every two weeks) plus axitinib versus sunitinib as first-line therapy in 886 patients with mRCC [126] showed superior ORR (55.2% vs. 25.5%, respectively) and PFS (13 vs. 8.4 months, respectively) across tumor PD-L1 status in patients receiving avelumab plus axitinib compared to those receiving sunitinib [126]. Overall, the rates of high-grade AEs were equivalent between the groups at 71.2% in the combination group and 71.5% in the sunitinib arm [126].

The second approved regimen involves the combination of pembrolizumab and axitinib [152]. Pembrolizumab combined with axitinib was better tolerated, and a phase Ⅰb study (NCT02853331) of treatment-naïve mRCC patients by Atkins et al. [154] reported a 65% rate of grade ≥ 3 AEs, and an impressive 73% ORR and 8% CR rate [154]. These encouraging results prompted KEYNOTE-426 (NCT02853331), a landmark phase III trial randomizing 1062 treatment-naïve patients with mRCC to pembrolizumab (200 mg every three weeks) plus standard doses of axitinib or sunitinib [152]. The KEYNOTE-426 trial in patients with mRCC reported improvements in PFS (HR, 0.69; *p* < 0.001) and OS (HR, 0.53; *p* < 0.0001) following treatment with this combination compared to that with sunitinib in the “intention-to-treat” population [152]. ORR, a secondary endpoint, was also significantly improved with axitinib plus pembrolizumab compared to that with sunitinib (59.3% vs. 35.7%, respectively; *p* < 0.0001) [152]. Updated results from this trial were recently presented with a median follow-up of 27 months, showing that combination therapy with pembrolizumab plus axitinib elicited durable responses that remained superior to sunitinib monotherapy [155]. These results prompted an accelerated FDA approval for the use of pembrolizumab and axitinib as first-line therapy for patients with mRCC [155].

The CheckMate 9ER trial comparing nivolumab plus cabozantinib to sunitinib in 651 previously untreated mRCC patients demonstrated significant benefits of nivolumab plus cabozantinib with respect to PFS, OS, and likelihood of response [156]. An objective response occurred in 55.7% patients receiving nivolumab plus cabozantinib and in 27.1% patients receiving sunitinib [156]. Overall, 19.7% patients in the combination group discontinued at least one of the trial drugs owing to AEs, and 5.6% discontinued both [156]. Lenvatinib, in combination with pembrolizumab or everolimus, is effective against mRCC (trial funded by Eisai and Merck Sharp and Dohme; CLEAR ClinicalTrials.gov number, NCT02811861) [157]. In this phase III trial, patients with mRCC and no previous systemic therapy were randomly assigned to receive lenvatinib plus pembrolizumab (355 patients), lenvatinib plus everolimus (357 patients), or sunitinib (357 patients) [157]. Lenvatinib plus pembrolizumab was associated with significantly longer PFS and OS than sunitinib [157]. Grade ≥ 3 AEs emerged or worsened during treatment in 82.4% patients receiving lenvatinib plus pembrolizumab, 83.1% patients receiving lenvatinib plus everolimus, and 71.8% patients receiving sunitinib [157]. Grade ≥ 3 AEs occurring in at least 10% patients in any group included hypertension, diarrhea, and elevated lipase levels [157].

### 6.3. Combination Strategies: ICI + Radiotherapy 

Recent preclinical studies have suggested a synergy between radiation and immunotherapies such as ICI [11,158,159,160,161] because radiotherapy evokes immunological changes in both the tumor and its microenvironment (by promoting effector immune cell recruitment), and induces systemic responses by promoting anti-tumor immunity (the “abscopal effect”) via several mechanisms, such as enhanced tumor antigen release, exposure to novel tumor antigens, increased immunogenic cell death, and increased pro-inflammatory cytokines that activate T cells [11,27,35,162,163,164].

The effects of radiation on tumor cells are primarily due to the generation of double-strand breaks that lead to the induction of different forms of cell death, including apoptosis, necrosis, autophagy, or mitotic catastrophe [11,27]. The discovery of immunogenic cell death (ICD) and abscopal effects provides formal evidence for the immunological effects of radiotherapy [27,165,166]. Abscopal effects describe the anti-tumor effects of radiotherapy in lesions outside the radiation field by triggering systemic anti-tumor effects [27,35,163]. The discovery of ICD as a molecularly defined process that leads to priming and activation of immune cells has recently led to a paradigm shift [27]. Radiotherapy has been shown to induce all three arms of the ICD and is therefore regarded as a potent inducer of ICD [162]. Radiation regimens that induce immunologically silent forms of cell death, that is, apoptotic cell death, are therefore not expected to synergize with ICB, while doses and fractionation that trigger inflammatory responses could be used as immunomodulators to induce the additive effects of radiotherapy and immunotherapy [167].

Radiation also upregulates MHC class I expression on the tumor cell surface to enable better antigen presentation of tumor-specific peptides for recognition by cytotoxic T cells, enhancing antigen presentation by MHC class I molecules [27,168,169,170]. Moreover, radiation-induced DNA damage can cause an increase in mutational load [171] and generate neoantigens that can be recognized and targeted by the immune system [172]. Radiation-induced DNA damage that causes leakage of DNA into the cytosol is known to be sensed by the stimulator of interferongens (STING), leading to the activation of innate and adaptive immune responses [27,173]. When cytosolic DNA is detected, the product of cyclic GMP–AMP synthase and cyclic GMP–AMP activates STING [27,173]. STING induces the transcription of type I interferon genes via a cascade that involves the STING downstream factors, tank-binding kinase, interferon regulatory factor 3, and nuclear factor kappa light chain enhancer of B cells [27,173,174].

Several studies have demonstrated that radiotherapy induces an increased influx of immune cells into the BM [27]. Radiation has profound effects on the secretion of cytokines that serve as chemoattractants for different immune cells, including DCs and macrophages [175]. In addition, radiation has been shown to affect key effector functions, such as phagocytosis, antigen presentation, and cytotoxicity, and alters the activation states of immune cells [176,177,178]. Therefore, exploiting the immunomodulatory functions of radiotherapy represents an attractive tool to convert immunologically cold environments into hot environments to increase the response rates of immunotherapy [27].

In addition to the roles of surgery and systemic therapies in the management of select patients with BM, radiotherapy remains an important cornerstone of treatment in most patients [17]. Whole brain radiation therapy (WBRT) was the standard treatment for most patients with BM because it could be administered easily, was widely available, provided symptom palliation, and treated both visible and occult lesions [17]. Although the recommendation of WBRT has become limited because of its neurotoxicity, it remains the most used treatment for patients with BM [17], particularly among those with multiple BM (>10 metastases) [17]. However, because of the biological characteristics of RCC that are resistant to conventional fractionation RT and WBRT-induced neurotoxicity, the use of focused radiation, such as SRS, is increasing for multiple BM, provided that the disease burden is limited [179,180]. SRS has a low rate of adverse radiation effects (AREs) relative to WBRT [181]. SRS also results in substantially less cognitive dysfunction and fatigue and can be delivered with minimal delay in secondary systemic therapies [17,182]. High-dose, single-fraction SRS should be considered for patients who are not candidates for surgical resection, depending on the number of BMs and prognosis [11].

Emerging combinations also include ICIs associated with radiotherapy [136]. Radiotherapy remodels intratumoral T cell responses and supports refined sequencing of combination strategies in RCC [183]. The specific observations of T cell expansion and contraction within distinct time points of observation offer a rationale for the timing of combination strategies that leverage endogenous T cell responses to improve patient outcomes [183]. RCC patients treated with SBRT exhibit broad transcriptional immune activation and increased clonality, with an underlying heightened proportion of dominant motifs [183]. Pathway analysis showed radiation-specific enrichment of immune-related processes, and T cell receptor sequencing revealed increased clonality in radiation-treated tumors [183]. Collectively, the data show that the dynamics of tumor-enriched clone expansion and contraction provide justification for single-dose radiation as an immune-sensitizing agent in RCC [183]. Overall, these results indicate robust intratumoral immune remodeling and a window of tumor-resident T cell expansion following radiation that may be leveraged for the rational design of combinatorial strategies [183].

Data on patients with mRCC were extracted from a retrospective international multicenter registry study, which investigated concurrent stereotactic radiotherapy (SRT) (≤30 days) with ICI therapy [184]. Fifty-three patients who underwent 128 sessions of SRT were included, of which 58% presented with oligometastatic disease [184]. Combined treatment immunotherapy and concurrent SRT were safe, without severe toxicity [184]. Full-dose SRT should be considered to achieve optimal metastasis control in patients receiving immunotherapy [184]. Favorable PFS and OS were observed in patients with oligometastatic RCC with a good ECOG-PS, which forms the basis for prospective testing of this treatment strategy in properly designed clinical trials [184]. Ahmed et al. [185] recently reported data from melanoma BM patients who received nivolumab plus SRS, demonstrating high rates of local BM control of 91% and 85% at the 6- and 12-month follow-up, respectively. A retrospective study of 75 melanoma patients with BM by Qian et al. [186] suggested that administering ICI within four weeks of SRS improves the local response compared to that with initiating treatment more than four weeks later. They concluded that immunotherapy can have a synergistic effect with radiosurgery in BM treatment, even in those not known to have PD-L1 expression, and that early local response is greater and more rapid with concurrent immunotherapy and SRS [186]. Eight studies [185,187,188,189,190,191,192] identified SRS using anti-PD-1/PD-L1 [181]. The treatment of SRS with a combination of anti-CTLA4 and anti-PD-1/PD-L1 did not increase neurotoxicity [181].

A few hurdles prevent higher response rates and more sustainable anti-tumor reactions in combination with radiotherapy [27]. Determining the optimal scheduling for radio-immunotherapy is a major challenge [27,193,194]. Possible regimens include concurrent, sequential, or neoadjuvant application of the treatment modules [27,195]. While there are several ongoing clinical trials that aim to compare the efficacy of ICIs in combination with either WBRT or SRS, there are only a few trials that are specifically designed to evaluate how different schedules affect the safety and efficacy of combined treatment [27]. The optimal schedule is tumor-type and immunotherapy-dependent [27]; however, to date, majority of trials report data that provide evidence for the benefit of concurrent schedules [186,196,197,198,199] and the lowest response rate if radiotherapy is administered after immunotherapy [27]. Concurrent ICI (within two weeks) was not associated with increased rates of immune-related adverse events or acute neurologic toxicity and predicted a decreased likelihood of developing ≥ 3 new BMs after SRS [198]. A comprehensive, study-level meta-analysis of BM treatments suggests that combinations of RT and ICI result in higher OS, yet comparable neurotoxicity profiles vs. RT alone, with the superiority of concurrent vs. sequential combination regimens [197].

A few hurdles prevent higher response rates and more sustainable anti-tumor reactions in combination with radiotherapy [27]. Determining the optimal scheduling for radio-immunotherapy is a major challenge [27,193,194]. Possible regimens include concurrent, sequential, or neoadjuvant application of the treatment modules [27,195]. While there are several ongoing clinical trials that aim to compare the efficacy of ICIs in combination with either WBRT or SRS, there are only a few trials that are specifically designed to evaluate how different schedules affect the safety and efficacy of combined treatment [27]. The optimal schedule is tumor-type and immunotherapy-dependent [27]; however, to date, majority of trials report data that provide evidence for the benefit of concurrent schedules [186,196,197,198,199] and the lowest response rate if radiotherapy is administered after immunotherapy [27]. Concurrent ICI (within two weeks) was not associated with increased rates of immune-related adverse events or acute neurologic toxicity and predicted a decreased likelihood of developing ≥ 3 new BMs after SRS [198]. A comprehensive, study-level meta-analysis of BM treatments suggests that combinations of RT and ICI result in higher OS, yet comparable neurotoxicity profiles vs. RT alone, with the superiority of concurrent vs. sequential combination regimens [197]. 

Different doses or fractionations are believed to induce different forms of cell death [162] and thus modulate downstream cellular responses [27]. It is critical to evaluate whether radiation dose and fractionation that is optimal to induce an immune response in the CNS is tolerated by the sensitive brain tissue [27]. Hypofractionation might not be favorable when combined with immunotherapy [27,158]. To optimize radiation dose and fractionation as well as scheduling for therapeutic applications, it is essential to investigate the molecular basis of the genotoxic and immune modulatory effects of radiotherapy [27]. Moreover, local and systemic immune responses can also be modulated by radiation-induced changes in different cell populations in the TME [27]. Direct and indirect effects on tumor cells and tumor-associated immune cells together determine the extent to which radiotherapy increases the immunogenicity of tumors and the synergy between radiotherapy and immunotherapy [27]. Since immunotherapies rely on functional T cells, their ablation or inactivation is expected to abrogate critical anti-tumor immune responses [27]. Conventional 2 Gy doses administered once daily can inactivate tumor-infiltrating T cells [27,200]. Systematic investigation of the dose dependency of immune responses directed against RCC-BM is crucial to determine optimal regimens to increase the immunogenicity of tumors and boost the immune system for effective anti-tumor responses that synergize with immunotherapy [27].

## 7. Future Perspective

Patients with RCC-BM have a poor prognosis, and they tend to be excluded from pivotal ICI trials, resulting in limited knowledge on the anti-tumor activity of immunotherapy in RCC-BM [11,16,35,47,133,201,202]. Additional reasons for this exclusion may depend on the large molecular size of ICIs, which limits their ability to cross the BBB, use of steroids to resolve symptomatic edema of BM, which may alter immune system activity, the risks of metastatic pseudoprogression and hyperprogression, and concerns that inflammatory responses might lead to neurological complications [11,16,35,47,133,201,202,203,204,205]. Modern registration trials of ICIs, including those for RCC, have therefore allowed the inclusion of asymptomatic BM patients in defined but rather small subcohorts [16].

Despite their clinical efficacy, ICIs can induce various immune-related AEs (irAEs), limiting their use in many patients [1,27,206,207]. ICIs may affect peripheral tolerance to autoantigens, resulting in autoantibody formation, which could be associated with irAEs in various organs [207]. Antigen sharing or cross reactivity leads to a T-cell-mediated response not only to tumor cells, but also to healthy cells [208]. ICIs can also release T-cells with subsequent production of proinflammatory cytokines such as interferon-γ and tumor necrosis factor, which may result in excessive off-tumor inflammation and autoimmunity [207]. Inflammatory responses are often associated with swelling, which would harm the delicate structures of the CNS and ultimately lead to brain damage [27]. Most irAEs tend to be mild and self-limiting; however, potentially life-threatening events can occur in a few severe cases (grade 3 or 4) [207,209]. Neurologic toxicities from ICIs, with an incidence of 1%–2%, include CNS paraneoplastic syndromes, encephalitis, multiple sclerosis, and hypophysitis [1]. Close follow-up and low thresholds for investigative work-ups are essential in ensuring that patients developing irAEs are promptly treated, such that any treatment interruption can be resumed in a timely manner [207,210]. Other known immunotherapy-related adverse events associated with ICIs include dermatitis (all-grade incidence of 17%), endocrinopathies (10%), colitis (2%), hepatitis (3%), and pneumonitis (3%) [1].

As the use of ICIs continue to expand, it will become more crucial that early detection and management of these irAEs becomes paramount to maximize the duration of treatment while minimizing toxicities for patients [207]. Early reports of symptoms can identify more irAEs with lower severity (grade 1 or 2), which could be treated with supportive measures without withholding ICI therapy [207]. Moreover, early diagnosis of irAEs may also prevent progression to higher levels of toxicity, which may subsequently prevent a patient from safely resuming treatment [207]. Although trials investigating BM have not shown higher rates of toxicities or neurologic adverse events compared to those with extracranial metastases, side effects have been observed. Therefore, until these comparisons are available, the approved combinations appear to have a distinct advantage, mainly in poor-risk or poor-leaning intermediate-risk diseases in which a robust response is desired [1].

Notably, the association between the development of irAEs and improved therapeutic efficacies is a dilemma faced by oncologists [207]. The development of moderate or severe irAEs can serve as a surrogate marker of response to ICIs [207,210]. A positive association is observed between the development of irAEs and response rates, time to treatment failure, and survival in patients treated with ICIs, irrespective of the disease site, type of ICI, and irAE [207,209,210,211,212,213]. Therefore, it is highly desirable to identify predictive biomarkers of both efficacy and toxicity associated with the use of ICIs, thereby facilitating guided treatment decisions [207].

A fraction of patients with BM benefit from treatment with ICI [214,215,216], and the degree and phenotype of immune cell infiltration have been used to predict the response to ICI [214]. The divergent results of immunotherapy in patients with BM allow for the data-driven design of novel therapeutic interventions [42,217,218]. CSF can provide fundamental information about the genomic characteristics of BM and hence can be used as a relatively non-invasive liquid biopsy [214,219,220]. A continuum of cellular T cell states indicates tumor reactivity and clonal expansion, which are also detectable in the CSF [214]. Importantly, inflammatory states that can predict ICI response, such as CD8+ T cell tumor infiltration, are recapitulated in CSF analysis [214]. CSF can recapitulate the immune landscape of the brain lesion, indicating that analysis of the CSF can provide critical information about the brain TME in a relatively non-invasive manner, as well as characterize and assess the degree of inflammation in brain lesions, which could be used to predict the response to ICIs [214]. Importantly, TCR clonotypes in the CSF match those of brain lesions, directly linking immune profiles from both compartments [214]. The CSF immune cell profile can facilitate the characterization of the immune TME in brain metastatic lesions and longitudinally monitor the evolution of the cancer immune response [214].

## Data Availability

Not applicable.
